# Quantitative EEG‐Based Detection of Poststroke Delirium Endotypes and Their Association With Biomarkers of Inflammation

**DOI:** 10.1002/brb3.71678

**Published:** 2026-09-06

**Authors:** Max Blücher, Nursena Armagan, Anna‐Christina Osswald, Annerose Mengel, Antje Vogelgesang, Johanna Ruhnau, Robert Fleischmann

**Affiliations:** ^1^ Department of Neurology University Medicine Greifswald Greifswald Germany; ^2^ Department of Neurology & Stroke University of Tübingen Tübingen Germany

**Keywords:** biomarkers, delirium, EEG, neuroinflammation, stroke

## Abstract

**Background and Purpose:**

Poststroke delirium (PSD) is common and prognostically relevant, yet under‐detected. Mechanistic and biomarker research is constrained by reliance on an intermittently observed binary phenotype, while delirium reflects one severity level on a continuum of delirium‐related encephalopathy. Quantitative EEG (qEEG) may capture encephalopathy endotypes more directly and enable severity‐spectrum characterization.

**Methods:**

In this prospective, single‐center, observational cohort study, 87 consecutive patients with acute ischemic stroke or transient ischemic attack were assessed within 48 h using the Confusion Assessment Method (CAM). A 64‐channel EEG was recorded at enrollment; spectral power (delta/theta/alpha/beta) and functional connectivity metrics (phase lag index; amplitude envelope correlation corrected [AECc]) were computed. Neuroinflammatory and systemic biomarkers were quantified from routine serum sampling in an exploratory, add‐on subcohort (*n* = 31).

**Results:**

PSD occurred in 28 of 87 (32%). PSD was characterized by spectral slowing and altered AECc. A multivariable qEEG model discriminated PSD with AUC = 0.892 (*p* < 0.001) and overall accuracy of 81.4% (non‐delirium 89.8%, delirium 63.0%). In the paired EEG plus serum subset, nominal exploratory correlations between qEEG metrics and selected biomarkers suggested links between network dysfunction and inflammatory signaling, including inverse correlations of theta‐band AECc with VILIP‐1 (*r* = −0.454, *p* = 0.045) and CX3CL1 (*r* = −0.604, *p* = 0.005), whereas biomarker–phenotype associations were less consistent.

**Conclusions:**

qEEG connectivity provides an encephalopathy‐proximal readout that can detect PSD and may characterize delirium‐related endotypes beyond the intermittently observed clinical phenotype. Findings require validation in larger, multicenter cohorts.

## Graphical abstract

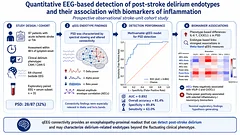

**Abbreviations**
AECc: amplitude envelope correlation corrected
CAM: Confusion Assessment Method
CAM‐ICU: Confusion Assessment Method for the Intensive Care Unit
NIHSS: National Institutes of Health Stroke Scale
PLI: phase lag index
PSD: poststroke delirium
RASS: Richmond Agitation–Sedation Scale
TIA: transient ischemic attack

## 1 Introduction

Delirium is an acute, fluctuating disturbance of attention and awareness that reflects acute brain dysfunction and is associated with adverse short‐ and long‐term outcomes (Wilson et al. 2020). In patients with acute stroke, poststroke delirium (PSD) is both common and clinically significant, with reported incidences of up to 40% within the first 72 h in some cohorts. Meta‐analytic evidence further demonstrates that PSD is independently associated with poorer outcomes, including increased short‐term mortality and worse functional recovery (Zhang et al. 2024). In addition to direct patient harm, PSD leads to longer hospital stays and a greater need for institutional care, thereby increasing long‐term dependency and healthcare resource utilization (Gan et al. 2025; Prüss et al. 2012).

Despite this burden, PSD remains under‐detected in routine care. Bedside screening predominantly captures the clinical phenotype—that is, observable behavior and symptom ratings assessed intermittently with structured screening instruments (Ely et al. 2001; Inouye et al. 2014). While these tools are essential, they are inherently limited by the fluctuating nature of delirium and by stroke‐specific challenges such as aphasia, impaired communication, sedation, neglect, or hypoactive presentations, all of which can result in missed or misclassified cases. Consequently, mechanistic research and biomarker discovery that rely primarily on binary “delirium present/absent” labels are constrained by measurement noise and by the fact that the phenotype is not a direct readout of the underlying biology (Boßelmann et al. 2019; Fleischmann et al. 2021; Serpa Neto and Slooter 2012).

A central conceptual advance is distinguishing between the clinical phenotype and the underlying endotype. Contemporary frameworks emphasize that delirium is the fluctuating clinical manifestation of an underlying acute encephalopathy, and that the phenotype therefore represents only a partial representation of the encephalopathic process (Kotfis et al. 2025). In this view, delirium lies on a continuum of encephalopathic brain dysfunction ranging from subsyndromal alterations to coma, with the binary phenotype capturing only one thresholded severity level on that spectrum (Oldham and Holloway 2020). This distinction matters because biomarkers and mechanisms are expected to align more closely with the encephalopathy endotype than with intermittent behavioral observations, particularly when the phenotype is attenuated or obscured by stroke deficits and clinical context (Kotfis et al. 2025).

Quantitative EEG (qEEG) offers a physiology‐based approach to assessing the endotype by directly measuring neural population dynamics with millisecond precision and quantifying large‐scale network dysfunction at the bedside (Hanna et al. 2023; van Montfort et al. 2019). Across etiologies, delirium has been linked to spectral slowing and disrupted connectivity, reflecting impaired network integration and altered oscillatory coordination (Hanna et al. 2023; van Montfort et al. 2019). In the context of PSD, recent studies underscore the potential of EEG as a biomarker closely linked to the underlying encephalopathy and highlight the need for diagnostic validation in real‐world stroke cohorts rather than relying solely on pathophysiological interpretation (van der et al. 2025). Notably, emerging evidence indicates that EEG‐derived markers of encephalopathy may correspond more closely with biological injury and inflammatory signaling than do clinical phenotype measures, supporting the idea that endotype‐focused approaches can enhance biomarker research (Klawitter et al. 2025).

The primary aim of this study is to validate previously reported qEEG features for identifying PSD by evaluating their ability to discriminate cases based on standardized clinical PSD assessments. In addition, we investigate whether systemic and neuroinflammatory serum biomarkers are associated with the clinical phenotype and the qEEG‐defined endotype, given heterogeneous and sometimes conflicting biomarker findings in delirium and PSD (Kowalska et al. 2018; Laichinger et al. 2023; Prüss et al. 2012; Wilson et al. 2020). We hypothesize that biomarker associations will be more consistent with the qEEG‐defined endotype than with the intermittently observed clinical phenotype, thereby offering a more informative approach for mechanistic inference and hypothesis generation.

## 2 Materials and Methods

### 2.1 Study Design and Setting

This prospective observational single‐center cohort study was conducted on the acute stroke‐unit at University Medicine Greifswald, Germany. The project comprised two sequential parts. First, a primary EEG study was implemented to acquire standardized bedside EEG recordings with delirium assessment under routine stroke‐unit conditions. This EEG acquisition phase started in January 2021 and continued for about 1 year until January 2022, reflecting the period in which trained personnel and equipment were consistently available to perform study examinations per protocol.

A second biomarker add‐on phase was initiated in February 2022 and continued until January 2023 to explore whether circulating biomarkers relate more consistently to physiology‐based EEG endotypes than to the binary clinical delirium phenotype, and to compare biomarker associations anchored to phenotype versus endotype. EEG acquisition remained identical in the add‐on phase, and EEG data from both phases were pooled for EEG‐based analyses. Serum biomarkers were only available in the add‐on phase. Recruitment and EEG acquisition therefore continued after the first EEG year to obtain an exploratory paired EEG plus serum subset including patients with and without PSD for whom both analyzable EEG data and retained serum were available. This subset was availability‐based and exploratory rather than a formally matched case‐control sample. Baseline characteristics of the paired subset are reported in Table 1.

**TABLE 1:** Baseline characteristics of the full qEEG cohort and the exploratory paired EEG plus serum subset. PSD indicates poststroke delirium classified using Confusion Assessment Method‐based (CAM‐S) bedside assessment supported by routine documentation and standardized chart review within 48 h of stroke‐unit admission. The paired EEG plus serum subset comprises participants from the biomarker add‐on phase with both analyzable qEEG and retained admission serum available for exploratory analyses. This subset was availability‐based and exploratory rather than a formally matched case‐control sample. Values are mean ± SD or *n* (%). National Institutes of Health Stroke Scale (NIHSS) and modified Rankin Scale (mRS) refer to admission scores. CAM‐S values are reported only for participants with PSD.

		**PSD**		**No PSD**	
**Characteristic**	**Cohort total (*N* = 87)**	**Full qEEG cohort (*n* = 28)**	**Paired EEG plus serum subset (*n* = 17)**	**Full qEEG cohort (*N* = 59)**	**Paired EEG plus serum subset (*N* = 14)**
**Age, years (mean ± SD)**	75.3 ± 9.6	80.3 ± 7.7	81.4 ± 7.3	73.0 ± 9.5	72.9 ± 9.1
**Male sex, *n* (%)**	47 (54.0%)	14 (50.0%)	13 (76.5%)	33 (55.9%)	4 (28.6%)
**Female sex, *n* (%)**	40 (46.0%)	14 (50.0%)	4 (23.5%)	26 (44.1%)	10 (71.4%)
**NIHSS at admission, mean ± SD**	10.1 ± 7.9	13.4 ± 8.8	11.4 ± 6.8	6.3 ± 4.4	6.7 ± 4.5
**mRS at admission, mean ± SD**	3.4 ± 1.1	3.8 ± 1.0	3.8 ± 0.8	2.9 ± 1.0	3.2 ± 0.8
**CAM‐S (long form), mean ± SD**	8.9 ± 2.3	8.9 ± 2.0	9.0 ± 2.7	—	—

### 2.2 Participants and Eligibility Criteria

Consecutive patients admitted with suspected acute ischemic stroke or transient ischemic attack (TIA) were screened. Patients were enrolled within 48 h of symptom onset, provided that study procedures could be completed without delaying acute stroke care.

Inclusion required a clinical presentation consistent with acute ischemic stroke or TIA with onset within 48 h of admission. Exclusion criteria were large territorial infarction, including large middle cerebral artery territory infarction, and cortical structural brain pathology not attributable to the index cerebrovascular event, such as brain tumor or traumatic brain injury. Patients requiring primary intensive care management, including mechanical ventilation, were not considered eligible for this stroke‐unit cohort because valid delirium assessment and standardized study EEG acquisition were not feasible under those conditions. Accordingly, the present cohort reflects a stroke‐unit population in whom standardized bedside EEG and delirium assessment were feasible, rather than the full spectrum of stroke severity. Patients with the most severe strokes requiring primary intensive care management and patients with large territorial infarctions were not represented. Generalizability is therefore strongest for patients with mild‐to‐moderate stroke‐unit presentations.

### 2.3 Regulatory Aspects and Data Collection

The study was approved by the institutional ethics review board of University Medicine Greifswald (BB 103/20; later amended for biomarker samples) and conducted in accordance with the Declaration of Helsinki. Written informed consent was obtained from all participants or their legal representatives prior to study‐specific procedures. If patients lacked capacity and no representative was available in time, a purely study‐driven EEG was not performed. Participation in serum biomarker analyses required additional consent, and biomarker availability also depended on retained admission serum from clinically indicated blood draws. Participants who declined EEG were excluded from EEG analyses. All study procedures were performed by delegated physicians or specially trained medical students to ensure standardization without disrupting routine care.

Demographic variables and medical history were collected from routine documentation and structured study records. Stroke syndrome was classified according to the Oxfordshire Community Stroke Project categories, and stroke etiology was classified using TOAST. Stroke severity and disability were assessed using the National Institutes of Health Stroke Scale (NIHSS) and modified Rankin Scale (mRS) during hospitalization. Treatment characteristics were documented, including reperfusion therapy and sedative exposure when relevant.

PSD was assessed at the bedside by trained study personnel using the Confusion Assessment Method (CAM). In addition, routine clinical documentation by the stroke‐unit team (including physician assessments) was reviewed, and a standardized post‐hoc chart review was performed to minimize missed cases (Fleischmann et al. 2021; Saczynski et al. 2014). Delirium symptom severity was quantified using CAM‐S long form (Inouye et al. 2014). Psychomotor subtype was classified as hyperactive, hypoactive, or mixed using Richmond Agitation–Sedation Scale (RASS) (Sessler et al. 2002). Assessments were performed by trained study personnel following standardized procedures. For the standardized study assessment, CAM‐based bedside evaluation was performed within the enrollment window, and study EEG was recorded once at enrollment as early as feasible within the 48‐h onset window. PSD case ascertainment was additionally supported by routine clinical documentation and standardized chart review in order to reduce missed fluctuating cases. Serum biomarker analyses were based on retained routine blood samples, with the present analyses focusing on the peak value within the first 24 h after admission. Accordingly, phenotype, EEG, and serum measures should be interpreted as early‐window measures that were clinically related but not necessarily perfectly time‐synchronous.

### 2.4 EEG Acquisition, Preprocessing, and Quantitative Feature Extraction

EEG was recorded once at enrollment using a 64‐channel system with waveguard electrodes and an eego amplifier (ANT Neuro, Enschede, The Netherlands). Recordings were obtained at the bedside as early as feasible within the 48‐h onset window. EEG data were imported into MATLAB and visually inspected. Preprocessing used FieldTrip for visualization, epoching, and manual rejection of artifactual epochs, with particular attention to eye movement and muscle artefacts (Oostenveld et al. 2011). Only artifact‐free epochs were retained. Power spectral density was computed using FFT‐based analysis for canonical frequency bands. Band power was expressed as relative power by dividing band‐limited power by total power across 0.5–30 Hz. Delta was defined as 0.5–4 Hz, theta as 4–8 Hz, alpha as 8–13 Hz, and beta as 13–30 Hz. For exploratory biomarker–EEG association analyses (Figure 2), we additionally extracted absolute total power (0.5–30 Hz), alpha subbands (alpha1, 8–10 Hz; alpha2, 10–13 Hz), and peak frequency, defined as the frequency of maximal power within 4–13 Hz. Phase‐based functional connectivity was quantified using Phase lag index (PLI) (Stam et al. 2007). Amplitude‐based coupling was quantified using amplitude envelope correlation (AEC) based on Pearson correlations of Hilbert‐derived amplitude envelopes (Bruns et al. 2000). A leakage‐corrected metric (AECc) was used to reduce spurious coupling from volume conduction. For amplitude coupling, the positive part of the correlation coefficient was used to summarize coupling strength on a 0–1 scale. Prespecified global summary measures for power and connectivity were derived for statistical testing.

### 2.5 Serum Sampling and Laboratory Assays

Serum biomarker sampling was implemented only in the biomarker add‐on phase. Biomarkers were obtained daily from retained surplus serum from clinically indicated blood draws within the first 7 days of admission, without additional venipuncture. Routine blood tests were generally and systematically available only on the day of admission and the morning of the next day. Therefore, the present analyses focus on the peak value within the first 24 h after admission, because later time points were not consistently available across patients. During the add‐on phase, recruitment was continued pragmatically to obtain a paired EEG plus serum subset including both delirious and non‐delirious participants with available analyzable EEG and retained admission serum. This subset was therefore availability‐based and exploratory rather than a formally matched case‐control sample. Baseline characteristics of the paired subset are reported separately in Table 1.

Samples were processed according to standard laboratory procedures and stored at −80°C until batch analysis. Thirteen analytes were quantified using a multiplex assay (BioLegend LEGENDplex Human Neuroinflammation Panel 1): VILIP‐1, MCP‐1 (also known as CCL2), sTREM‐2, BDNF, free active TGF‐β1, VEGF, IL‐6, sTREM‐1, β‐NGF, IL‐18, TNF‐α, sRAGE, and CX3CL1 (also known as fractalkine). Measurements followed manufacturer's instructions using a modified protocol with reduced consumables.

### 2.6 Sample Size Considerations and Statistical Analysis

The EEG cohort was an exploratory convenience sample accrued during the predefined EEG acquisition period, with an expected sample size of about 100 participants within 1 year under comparable stroke‐unit constraints and enrollment within 48 h of onset. The biomarker add‐on phase required a longer recruitment window to assemble a matched paired EEG plus serum subset because serum availability depended on routine blood draws and additional consent.

Patients were classified as PSD or NoPSD based on CAM. Analyses were complete‐case within each modality, with EEG analyses in the pooled EEG cohort, serum analyses in the serum subcohort, and EEG plus serum association analyses in the paired subset. Normality was assessed using Shapiro–Wilk test and variance homogeneity using Levene's test where applicable. Between‐group comparisons used independent samples *t* tests for approximately normal data and Mann–Whitney *U* tests otherwise, with effect sizes reported as Cohen's d and *r*, respectively. Correlations used Spearman rank coefficients, including within‐group correlations among qEEG measures, correlations among serum biomarkers in the serum subcohort, qEEG biomarker correlations in the paired subset, and correlations with delirium severity using CAM‐S.

Receiver operating characteristic (ROC) analyses were performed for EEG measures with significant group differences, and AUC with 95% confidence intervals was reported. Logistic regression results were reported as odds ratios with 95% confidence intervals, and a multivariable EEG model for PSD discrimination was derived using backward conditional binary logistic regression based on EEG variables significant in univariable testing. Model performance was summarized using Hosmer–Lemeshow goodness of fit, Nagelkerke *R* ^2^, overall classification accuracy, class specific accuracy, sensitivity, specificity, and ROC AUC. No class weighting, resampling, or threshold optimization was applied; classification metrics were calculated at the conventional default predicted‐probability threshold of 0.5.

Phenotype‐biomarker associations were assessed by comparing serum biomarker concentrations between PSD and NoPSD using two‐sided unpaired *t*‐tests when Shapiro–Wilk test supported approximate normality, applying Welch correction for unequal variances, and using two‐sided Mann–Whitney *U* tests otherwise, while CAM‐S biomarker associations used Spearman's rank correlation. Endotype biomarker associations were assessed in the paired EEG plus serum subset using Spearman correlations between qEEG metrics and serum biomarkers without assuming normality and were summarized in a correlation heatmap. Statistical significance was set at *p *< 0.05 with two‐sided testing, and analyses were performed using SPSS version 30. Because biomarker group comparisons and biomarker–qEEG correlations were exploratory and involved multiple tests, these analyses are reported as nominal, uncorrected findings intended for hypothesis generation rather than confirmatory inference. In addition, we performed a Benjamini–Hochberg false discovery rate sensitivity analysis for the exploratory phenotype‐anchored biomarker group comparisons. As a further sensitivity analysis, we identified the subset of participants for whom raw qEEG feature data and raw biomarker data could be linked. In this subset, Spearman correlations between qEEG measures and biomarkers were recalculated, and Benjamini–Hochberg false discovery rate correction was applied across the full family of tested associations.

## 3 Results

### 3.1 Cohort and Clinical Characteristics

A total of 87 participants had analyzable qEEG recordings and were included. Within this selected stroke‐unit cohort, PSD was identified in 28 of 87 patients (32%). Baseline characteristics of the EEG‐only and EEG plus serum cohorts are shown in Table 1.

Patients with PSD were older than those without PSD. Mean age was 80.26 = 7.67 years in PSD and 72.98 = 9.54 years in NoPSD (*p* < 0.001). Sex distribution was similar between groups, with 14/28(50.0%) male in PSD and 33/59(55.9%) male in NoPSD (*p* = 0.650). NIHSS at admission was higher in PSD (13.4 = 8.8) than NoPSD (6.3 = 4.4, *p* < 0.001). Admission mRS was higher in PSD (3.8 = 1.0) than NoPSD (2.9 = 1.0, *p* < 0.001). Among patients with PSD, 13 had a mixed psychomotor subtype, 8 were hypoactive, and 7 were hyperactive.

### 3.2 Between‐Group qEEG Differences and Discrimination Relative to Standardized Clinical Assessment

Between‐group comparisons revealed significant PSD‐related differences in spectral power and amplitude‐based coupling. Key qEEG group differences are summarized below, and a complete overview of prespecified qEEG metrics and their discriminative performance relative to standardized clinical assessment is provided in Table 2. In univariable analyses, delta power was higher in PSD than in NoPSD (0.751 ± 0.069 vs. 0.681 ± 0.090; *p* < 0.001), while alpha power and beta power were lower in PSD (0.030 ± 0.012 vs. 0.050 ± 0.028; *p* < 0.001 and 0.045 ± 0.0205 vs. 0.068 ± 0.0279; *p* < 0.001, respectively). Amplitude‐based coupling was also higher in PSD across multiple bands (AECc alpha: 0.172 ± 0.087 vs. 0.111 ± 0.027; *p* < 0.001, AECc theta: 0.175 ± 0.078 vs. 0.132 ± 0.038; *p* = 0.004, and AECc beta: 0.168 ± 0.0825 vs. 0.094 ± 0.0237; *p* < 0.001).

**TABLE 2:** qEEG markers and discriminative performance relative to standardized clinical PSD assessment. Relative band power is shown for delta (0.5–4 Hz), theta (4–8 Hz), alpha (8–13 Hz), and beta (13–30 Hz). AECc denotes leakage‐corrected amplitude‐envelope correlation. AUC values are reported with clinically assessed PSD as the positive class; for parameters lower in PSD, AUCs were direction‐corrected (AUC = 1 − original ROC AUC). Final model coefficient *p*‐values correspond to the two predictors retained in the backward conditional logistic regression model (AECc beta and AECc theta). The final multivariable model showed an AUC of 0.892 (95% CI 0.81–0.98) relative to standardized clinical PSD assessment. At the default predicted‐probability threshold of 0.5, the final model showed 89.8% specificity for NoPSD and 63.0% sensitivity for PSD.

EEG parameter	PSD (mean ± SD)	No PSD (mean ± SD)	Univariable *p*‐value	Univariable AUC	Final model coefficient *p*‐value	Final model AUC
**Relative delta power**	0.751 ± 0.069	0.681 ± 0.090	< 0.001	0.718	—	—
**Relative theta power**	0.140 ± 0.039	0.157 ± 0.048	0.123	0.608	—	—
**Relative alpha power**	0.030 ± 0.012	0.050 ± 0.028	< 0.001	0.754	—	—
**Relative beta power**	0.045 ± 0.021	0.068 ± 0.028	< 0.001	0.764	—	—
**AECc delta**	0.140 ± 0.059	0.116 ± 0.023	0.199	0.601	—	—
**AECc theta**	0.175 ± 0.078	0.132 ± 0.038	0.004	0.696	0.046 (−)	0.892
**AECc alpha**	0.172 ± 0.087	0.111 ± 0.027	< 0.001	0.747	—	
**AECc beta**	0.167 ± 0.083	0.094 ± 0.024	< 0.001	0.843	< 0.001 (+)	

**FIGURE 1:**
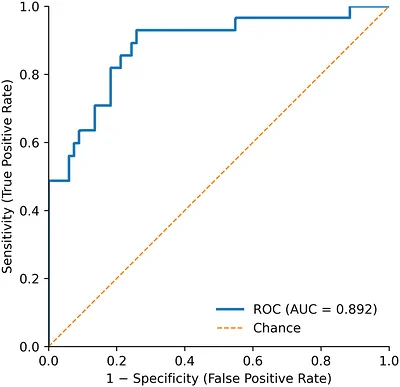
qEEG model discrimination relative to standardized clinical PSD assessment. Receiver operating characteristic (ROC) curve for the multivariable qEEG model distinguishing patients with clinically assessed poststroke delirium (PSD) from those without clinically assessed PSD (NoPSD) in the full qEEG cohort (*n* = 87). PSD was classified within 48 h using Confusion Assessment Method‐based bedside assessment supported by routine documentation and standardized chart review. The final model was derived by backward conditional binary logistic regression from qEEG variables showing significant univariable group differences and retained theta‐band amplitude envelope correlation corrected (AECc theta) and beta‐band amplitude envelope correlation corrected (AECc beta) as predictors. The ROC curve depicts sensitivity versus 1 − specificity across thresholds of the model‐predicted probability of clinically assessed PSD. Area under the curve (AUC) = 0.892 (95% CI 0.81–0.98; *p* < 0.001). The dashed diagonal line indicates chance‐level discrimination. AECc, amplitude envelope correlation corrected; AUC, area under the curve; NoPSD, no clinically assessed poststroke delirium; PSD, clinically assessed poststroke delirium; qEEG, quantitative electroencephalography; ROC, receiver operating characteristic.

To examine whether qEEG connectivity markers differentiated PSD from NoPSD, a binary logistic regression with backward stepwise selection was fitted, including those qEEG variables that showed significant group differences in univariable analyses. The final model retained two predictors. In the multivariable model, AECc beta was positively associated with PSD (*p* < 0.001), whereas AECc theta was inversely associated with PSD (*p* = 0.046). Although AECc theta was higher in PSD in univariable testing, its adjusted association became inverse in the multivariable model, consistent with shared variance between connectivity predictors and a suppression effect after adjustment. The model demonstrated a significant improvement in fit over the intercept‐only model (*χ* ^2^ = 42.730, *p* < 0.001), explaining 55% of the variance (pseudo‐*R* ^2^ = 0.55). It achieved an overall accuracy of 81.4%. At the default predicted‐probability threshold of 0.5, specificity for NoPSD was 89.8% and sensitivity for PSD was 63.0%. Discrimination in the ROC analysis was good relative to standardized clinical assessment (AUC = 0.892; 95% CI: 0.81–0.98; *p* < 0.001; Figure 1). In an exploratory sensitivity analysis adjusting the final qEEG model for age, discrimination remained similar (AUC = 0.887; bootstrap 95% CI 0.79–0.96). In this adjusted model, AECc beta remained independently associated with PSD (*p* = 0.0002), whereas the adjusted association of AECc theta was attenuated (*p* = 0.113).

### 3.3 Serum Biomarkers and PSD Phenotype

These analyses were restricted to the exploratory, paired EEG plus serum subset and should therefore be interpreted as subset‐based, availability‐dependent analyses rather than as estimates from the full EEG cohort. Significant between‐group differences were observed for IL‐6 and CX3CL1. IL‐6 was higher in PSD than NoPSD (83.23pg/mL, 95% CI: 47.97–118.50 vs. 41.30pg/mL, 95% CI: 26.73–55.88, *p* = 0.029). CX3CL1 was lower in PSD than in NoPSD (559.90pg/mL, 95% CI: 389.30–730.40 vs. 885.50pg/mL, 95% CI: 602.70–1168.00, *p* = 0.038). All other serum biomarker group comparisons were not significant. In a Benjamini–Hochberg false discovery rate sensitivity analysis across the exploratory phenotype‐anchored biomarker group comparisons, no biomarker remained statistically significant after correction.

### 3.4 Exploratory Serum Biomarker Associations With qEEG Measures

**FIGURE 2:**
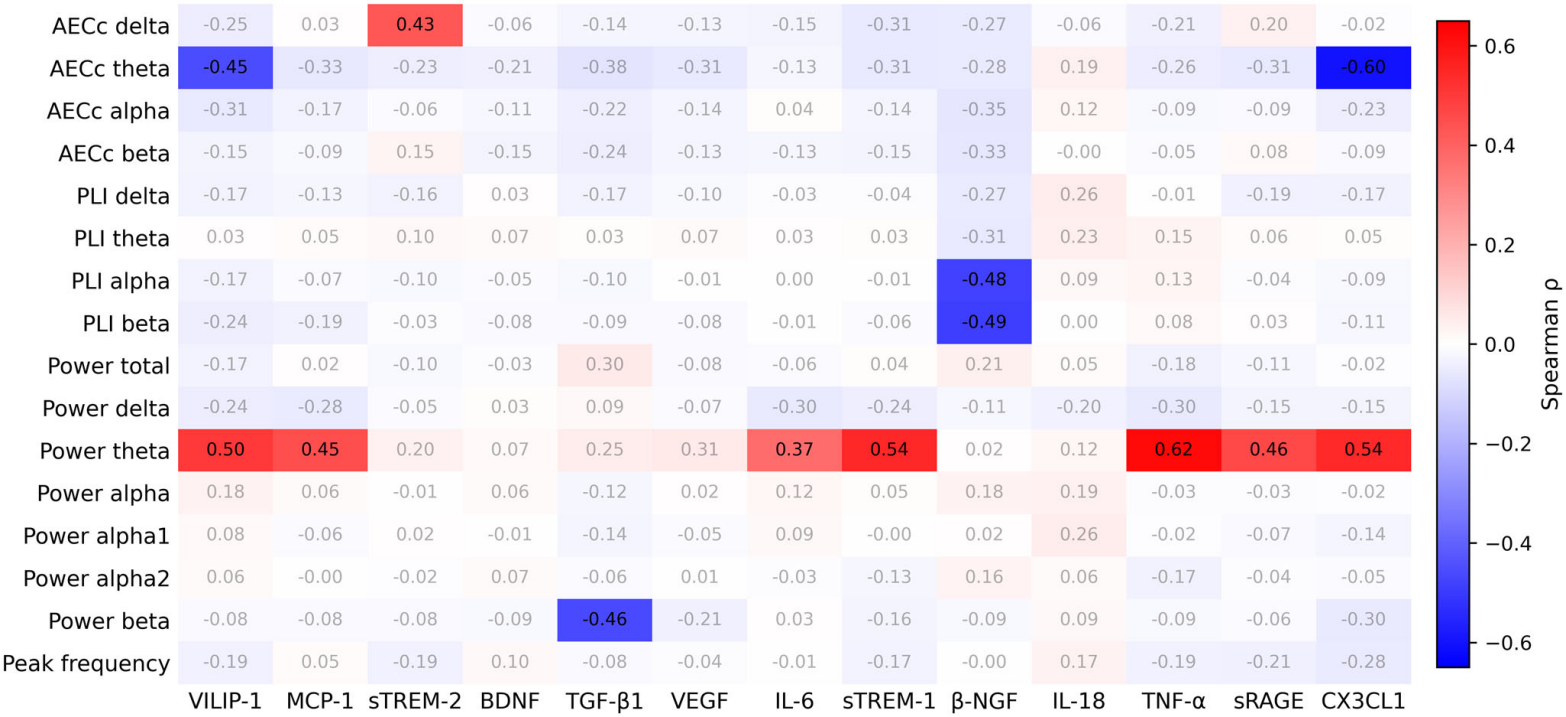
Exploratory correlations between serum biomarkers and qEEG measures. Heatmap of Spearman rank correlations (*ρ*) between serum biomarkers and qEEG measures in the paired EEG plus serum subset (*n* = 31). Biomarkers were quantified from routine serum sampling. qEEG measures include global spectral power, phase‐based connectivity (PLI), amplitude‐based coupling (AECc), and peak frequency. Cell values show *ρ* rounded to two decimals. Nominally significant correlations (two‐sided *p* < 0.05, uncorrected for multiple testing) are displayed with full opacity, whereas non‐significant correlations are rendered semi‐transparent. This figure is provided for hypothesis generation only. AECc, amplitude envelope correlation corrected; BDNF, brain‐derived neurotrophic factor; CAM‐S, Confusion Assessment Method–Severity; CX3CL1, fractalkine; IL, interleukin; MCP‐1/CCL2, monocyte chemoattractant protein 1; NGF, nerve growth factor; PLI, phase lag index; qEEG, quantitative electroencephalography; sRAGE, soluble receptor for advanced glycation end products; sTREM, soluble triggering receptor expressed on myeloid cells; TGF‐β1, transforming growth factor beta 1; TNF‐α, tumor necrosis factor alpha; VEGF, vascular endothelial growth factor; VILIP‐1, visinin‐like protein 1.

In the paired EEG plus serum subset, several nominal, uncorrected correlations were observed between qEEG measures and serum biomarkers (Figure 2). These analyses were exploratory and intended for hypothesis generation rather than confirmatory inference. Theta‐band measures showed the most consistent nominal associations, including negative correlations of theta AECc with VILIP‐1 (*r* = −0.454, *p* = 0.045) and CX3CL1 (*r* = −0.604, *p* = 0.005), and positive correlations of theta power with VILIP‐1 (*r* = 0.501, *p* = 0.024), MCP‐1 (*r* = 0.448, *p* = 0.048), sTREM‐1 (*r* = 0.544, *p* = 0.013), TNF‐α (*r* = 0.624, *p* = 0.003), sRAGE (*r* = 0.465, *p* = 0.039), and CX3CL1 (*r* = 0.541, *p* = 0.014). Additional nominal associations included negative correlations of alpha PLI with β‐NGF (*r* = −0.480, *p* = 0.032), beta PLI with β‐NGF (*r* = −0.489, *p* = 0.029), and beta power with TGF‐β1 (*r* = −0.458, *p* = 0.042). Given the multiplicity of tested biomarker–qEEG associations and the exploratory character of this analysis, these nominal correlations are interpreted strictly as hypothesis‐generating findings.

As an additional sensitivity analysis, we examined the raw data‐overlap subset of qEEG feature data and biomarker data. In this subset, several nominal associations were observed, including delta power with VILIP‐1 (*ρ* = −0.729, *p* = 0.0109), beta PLI with VILIP‐1 (*ρ* = −0.711, *p* = 0.0142), theta power with VILIP‐1 (*ρ* = 0.683, *p* = 0.0204), beta power with VILIP‐1 (*ρ* = 0.679, *p* = 0.0216), and theta power with MCP‐1 (*ρ* = 0.664, *p* = 0.0260). However, after Benjamini–Hochberg false discovery rate correction across the full family of qEEG–biomarker tests, no association remained statistically significant.

## 4 Discussion

In this prospective stroke‐unit cohort, PSD was frequent, affecting 28 of 87 patients (32.18%). qEEG measures differentiated PSD from NoPSD, with the strongest signal arising from connectivity‐based features and a multivariable EEG model that achieved excellent discrimination (AUC = 0.892). In parallel, a serum biomarker add‐on explored whether circulating inflammatory and neuroinflammatory markers relate more consistently to EEG‐based endotypes than to the binary clinical PSD phenotype. In the serum subcohort, IL‐6 and CX3CL1 showed nominal PSD‐related group differences, although these did not remain statistically significant after false discovery rate correction. The paired EEG plus serum dataset revealed multiple nominal correlations between qEEG features and biomarkers, particularly involving theta‐band measures and CX3CL1, which should be interpreted as hypothesis‐generating. These findings support the feasibility of bedside qEEG as an encephalopathy‐proximal readout in acute stroke and suggest that endotype‐anchored biomarker work could advance PSD pathophysiology beyond a phenotype‐only framework (Bowman et al. 2023; Kotfis et al. 2025; Slooter et al. 2020; Wilson et al. 2020).

### 4.1 PSD as a Spectrum Disorder and EEG‐Based Discrimination

The EEG model's AUC of 0.892 indicates excellent discrimination of clinically diagnosed PSD under routine stroke‐unit conditions and compares favorably with many risk prediction approaches that rely on clinical variables alone (Fleischmann et al. 2021; Oldenbeuving et al. 2011; Shi et al. 2012; Zhang et al. 2024). This diagnostic performance is clinically meaningful because PSD screening depends on intermittent bedside assessments that can miss fluctuating symptoms, and in stroke it is further challenged by aphasia, neglect, reduced arousal, and sedation (Fleischmann et al. 2021; Slooter et al. 2020; Wilson et al. 2020). At the same time, because PSD and NoPSD differed substantially in age and stroke severity, the present model cannot be interpreted as proving complete specificity for delirium independent of stroke burden. Rather, it should presently be understood as identifying PSD under real‐world stroke‐unit conditions. In an exploratory age‐adjusted sensitivity analysis, model discrimination remained similar, and AECc beta remained independently associated with PSD, but residual confounding by stroke severity remains possible.

From a conceptual perspective, however, assigning diagnostic properties to qEEG against a binary PSD phenotype remains inherently confounded because delirium is increasingly framed as the fluctuating clinical manifestation of acute encephalopathy rather than the encephalopathy itself (Kotfis et al. 2025; Slooter et al. 2020). In this framework, the phenotype represents a partial representation of an underlying severity spectrum that ranges from subsyndromal alterations through overt delirium to coma, and the same biological process may be present without meeting a categorical delirium threshold at a given assessment time (Bowman et al. 2023; Slooter et al. 2020; Wilson et al. 2020).

Against this background, the present results can be read in two complementary ways. First, the primary practical requirement was to show that qEEG can detect PSD cases that are identified by standardized clinical assessment in a real‐world stroke cohort, and the near 0.9 AUC supports this goal. Second, qEEG may also detect encephalopathy‐range network dysfunction in some patients who do not meet the binary phenotype criteria at the time of bedside assessment, which would appear as false positives when the phenotype is treated as the sole reference standard (Kotfis et al. 2025; Slooter et al. 2020). This possibility is consistent with consensus terminology and with evidence that electrophysiological signatures track acute brain dysfunction across etiologies, including delirium, in a state‐dependent manner (Hanna et al. 2023; van der et al. 2025; Wiegand et al. 2022; Wilson et al. 2020). It also aligns with emerging work that argues for moving from syndrome labels toward physiology‐grounded endotypes when aiming to understand mechanisms and develop targeted interventions (Bowman et al. 2023; Kotfis et al. 2025).

At the feature level, PSD was associated with higher delta power and lower alpha and beta power, which fits the broader delirium literature linking encephalopathy to spectral slowing and reduced faster activity (van der et al. 2025; Wiegand et al. 2022; Wilson et al. 2020). Beyond power, amplitude‐based coupling (AECc) was higher in PSD across the alpha, theta, and beta bands, with AECc beta and AECc theta retained as independent predictors in the final model. Altered connectivity is a recurring theme in delirium mechanistic work and qEEG reviews, although specific directionality can depend on metric choice, frequency band, and clinical context (Fleischmann et al. 2025; Hanna et al. 2023; van der et al. 2025; van Montfort et al. 2019; Wiegand et al. 2022). In stroke, focal lesions can introduce regional slowing that may contaminate global summary measures, which is why large territorial infarctions were excluded to reduce the likelihood that the observed effects simply reflect dominant regional injury patterns. Even with this design choice, future studies should explicitly test whether incorporating hemispheric asymmetry, such as ipsilateral versus contralateral spectral and connectivity features, improves discrimination and clarifies the contribution of focal lesion effects versus superimposed global encephalopathy (Mintz et al. 2024). This will be particularly important when extending qEEG approaches to more severe strokes and intensive care settings where both focal injury and systemic drivers of encephalopathy can be pronounced (Slooter et al. 2020; Wilson et al. 2020).

### 4.2 Serum Biomarker Associations With the PSD Phenotype and Endotype

Phenotype‐anchored biomarker comparisons in the serum subcohort showed nominal PSD‐related group differences for IL‐6 and CX3CL1, but these did not remain statistically significant after false discovery rate correction. This pattern mirrors the broader delirium biomarker literature where inflammatory signals, including IL‐6, often show associations but with substantial heterogeneity across cohorts, timing, and assay platforms, and along with inconsistent reproducibility in stroke‐specific settings (Kowalska et al. 2018; Lozano‐Vicario et al. 2023; Ruhnau et al. 2023). In this context, the nominal phenotype‐anchored biomarker differences observed in the present study should not be read as confirmatory biomarker evidence, but rather as hypothesis‐generating signals within a small, exploratory serum subset. The lack of significance after false discovery rate correction is consistent with the challenges inherent in anchoring biology to a binary and fluctuating clinical syndrome label, especially with small sample sizes and routine care sampling constraints (Ruhnau et al. 2023; Slooter et al. 2020; Wilson et al. 2020).

Two aspects are noteworthy for interpretation and hypothesis generation. First, the contrast between sparse, nominal phenotype‐anchored biomarker group differences and the broader pattern of nominal, endotype‐anchored EEG biomarker correlations is compatible with the notion that physiology‐based measures may sit closer to the neurobiological substrate of delirium‐related encephalopathy than binary phenotype labels do, but this interpretation remains exploratory (Klawitter et al. 2025; Kotfis et al. 2025; Slooter et al. 2020). Related work in critical care populations similarly suggests that EEG‐derived indices of brain dysfunction can show clearer biological coupling with circulating markers than symptom‐based delirium classifications, supporting the rationale for endotype‐first biomarker discovery strategies (Klawitter et al. 2025). Second, CX3CL1 illustrates why phenotype‐only interpretation can be misleading. The Day 0 group difference showed lower CX3CL1 in PSD, which is not an intuitively straightforward direction for a neuroimmune signaling axis; however, theta power correlated positively with CX3CL1 in the paired analyses, supporting a link between network slowing and inflammatory activation. CX3CL1 is a key chemokine in neuron–microglia communication and neuroinflammatory regulation, and peripheral levels may reflect complex, state‐dependent dynamics rather than a monotonic increase with clinical syndrome presence (Pawelec et al. 2020; Subbarayan et al. 2022; Xiao et al. 2023). Beyond biomarker‐specific interpretations, the observed biomarker–qEEG associations are biologically plausible within immune‐to‐brain signaling frameworks (Leunig et al. 2025). Peripheral inflammatory mediators can modulate neurocircuits and behavior via endothelial and brainstem pathways and contribute to inflammation‐related cognitive and behavioral changes. In parallel, inflammatory cues can promote myeloid cell recruitment and neuroimmune interactions that influence synaptic function, neuronal excitability, and cognition in systemic inflammatory states. Within this framework, qEEG signatures of encephalopathy may capture a physiologically meaningful neuroimmune endotype that is only partially reflected by the intermittently observed binary bedside phenotype. The present pattern therefore supports further longitudinal sampling aligned to repeated qEEG and repeated phenotype assessments (Ruhnau et al. 2023; Slooter et al. 2020; Wilson et al. 2020).

### 4.3 Relation to Previous Work and Implications for PSD Pathophysiology Research

Our qEEG findings align with a substantial body of delirium research linking acute brain dysfunction to slowing and altered network integration, and they extend this work by providing a real‐world diagnostic validation step in acute stroke. Reviews and multicenter studies report that delta increases and alpha and beta reductions are robust markers of delirium across etiologies, while connectivity changes vary by metric and band but consistently point to disturbed large‐scale integration (Hanna et al. 2023; van der et al. 2025; Wiegand et al. 2022; Wilson et al. 2020). The strong AUC in this cohort suggests that even a single, standardized bedside EEG may carry clinically relevant information about encephalopathy burden early after stroke. This matters because PSD is associated with worse outcomes and higher mortality, and improving detection could be a prerequisite for both prevention and mechanistic studies that aim to link biology to clinically meaningful trajectories (Fleischmann et al. 2021; Shi et al. 2012; Zhang et al. 2024).

Conceptually, the present results reinforce that PSD research may be accelerated by shifting from phenotype‐constrained designs toward endotype‐anchored designs. The Delirium Subtyping Initiative and related terminology work emphasize that one‐size‐fits‐all syndrome labels are unlikely to capture mechanistic heterogeneity and that physiology‐grounded measures are needed to define subtypes and targets (Bowman et al. 2023; Kotfis et al. 2025; Slooter et al. 2020). The biomarker add‐on in this study provides a hypothesis‐generating proof of concept that nominal correlations between circulating biomarkers and qEEG features can be explored even in a small, paired dataset, supporting the feasibility of larger prospective studies with prespecified endotype and biomarker endpoints. This approach may help reconcile why prior PSD biomarker studies have been inconsistent, because they often anchor analyses to a binary phenotype that is intermittently measured and only partially reflects the encephalopathy spectrum (Kowalska et al. 2018; Ruhnau et al. 2023; Slooter et al. 2020).

### 4.4 Limitations and Future Directions

Several limitations should be considered. First, this was a single‐center exploratory study with modest sample size, and the EEG model was derived and evaluated within the same cohort, so performance may be optimistic without external validation or cross‐validation. In addition, PSD prevalence in the cohort was 32%, and model classification performance was not symmetrical across classes since specificity for NoPSD was higher than sensitivity for PSD. This class imbalance is clinically relevant because overall accuracy can overstate model performance when the majority class is classified more accurately than the minority class.

Second, PSD and NoPSD groups differed substantially in age and stroke severity measures such as NIHSS and mRS, which may confound both qEEG and biomarker signals because more severe strokes are both more likely to produce PSD and more likely to produce global physiological disruption and systemic inflammatory responses (Oldenbeuving et al. 2011; Shi et al. 2012; Wilson et al. 2020; Zhang et al. 2024). This is an intrinsic challenge in PSD research because encephalopathy risk and stroke burden are tightly coupled, and controlling for severity can remove variance that may itself be mechanistically relevant to delirium. For this reason, we interpret the present qEEG model primarily as identifying PSD under real‐world stroke‐unit conditions rather than as proving complete specificity for delirium independent of stroke severity. In an exploratory sensitivity analysis adjusting the final model for age, model discrimination remained similar and AECc beta remained independently associated with PSD, but residual confounding by stroke severity remains possible.

Third, delirium assessment relied on intermittent, CAM‐based screening within a fluctuating syndrome, which can lead to misclassification, particularly for hypoactive states and in patients with communication deficits; this directly affects estimates of diagnostic performance for any comparator biomarker (Fleischmann et al. 2021; Slooter et al. 2020; Wilson et al. 2020). Fourth, qEEG was recorded once early after admission, which may not coincide with peak delirium expression in a fluctuating course, and repeated or continuous EEG could better capture temporal dynamics of encephalopathy. Fifth, serum biomarkers were added later as an exploratory endpoint; serum availability depended on retained routine samples and additional consent, and systematic routine sampling was most consistently available at Day 0 and Day 1, limiting temporal resolution and reducing power for biomarker analyses.

Finally, given the exploratory nature and the number of correlation tests, the biomarker analyses are vulnerable to false‐positive findings. We therefore report them as nominal, hypothesis‐generating results and performed a Benjamini–Hochberg false discovery rate sensitivity analysis for the phenotype‐anchored biomarker group comparisons. We also conducted an additional, raw data‐overlap sensitivity analysis for qEEG–biomarker correlations, in which no association remained statistically significant after false discovery rate correction. Even with these steps, the present biomarker findings require replication in larger cohorts.

These limitations define clear next steps. Future studies should predefine endotype and phenotype endpoints, use repeated delirium assessments and repeated qEEG, incorporate hemispheric asymmetry features to address focal lesion effects, and validate prediction models externally with appropriate calibration metrics. Biomarker work should adopt longitudinal sampling aligned to the encephalopathy timeline, and should prioritize designs that test whether biomarker associations strengthen when anchored to continuous qEEG endotypes rather than to binary syndrome labels.

### 4.5 Conclusion

This study supports qEEG as a feasible approach to characterizing PSD endotypes, with good discrimination against standardized clinical assessment in an acute stroke cohort. The serum add‐on suggests that biomarker associations may be more informatively explored when anchored to EEG‐defined endotypes than when anchored only to the binary PSD phenotype, motivating endotype‐first biomarker strategies in future PSD research. While exploratory and limited by sample size, a single‐center design, and routine sampling constraints, these data provide a rationale for prospective, multicenter validation and for mechanistic studies that integrate repeated qEEG with longitudinal biomarker profiling across the acute encephalopathy spectrum.

## Author Contributions

**Max Blücher**: conceptualization, data acquisition, data curation, formal analysis, methodology, validation, visualization, writing – original draft, writing – review and editing. **Nursena Armagan**: data curation, data acquisition, formal analysis, methodology, validation, visualization, writing – original draft, writing – review and editing. **Anna‐Christina Osswald**: data acquisition, data curation, investigation, project administration, validation, writing – review and editing. **Annerose Mengel**: conceptualization, methodology, supervision, validation, writing – review and editing. **Antje Vogelgesang**: investigation, methodology, resources, validation, writing – review and editing. **Johanna Ruhnau**: conceptualization, methodology, resources, supervision, validation, writing – review and editing. **Robert Fleischmann**: conceptualization, formal analysis, methodology, project administration, resources, supervision, validation, visualization, writing – original draft, writing – review and editing. All authors read and approved the final version of the manuscript and agree to be accountable for all aspects of the work.

## Funding

The authors have nothing to report.

## Ethics Statement

The study was approved by the institutional ethics review board of University Medicine Greifswald (BB 103/20; later amended for biomarker samples) and was conducted in accordance with the Declaration of Helsinki.

## Consent

Written informed consent was obtained from all participants or their legal representatives prior to any study‐specific procedures.

## Conflicts of Interest

The authors declare no conflicts of interest.

## Data Availability

De‐identified data supporting the findings of this study are available from the corresponding author upon reasonable request, subject to institutional approvals and applicable data protection regulations.
